# The Association between Insertion Sequences and Antibiotic Resistance Genes

**DOI:** 10.1128/mSphere.00418-20

**Published:** 2020-09-02

**Authors:** Mohammad Razavi, Erik Kristiansson, Carl-Fredrik Flach, D. G. Joakim Larsson

**Affiliations:** a Centre for Antibiotic Resistance Research (CARe), University of Gothenburg, Gothenburg, Sweden; b Department of Infectious Diseases, Institute of Biomedicine, The Sahlgrenska Academy, University of Gothenburg, Gothenburg, Sweden; c Department of Mathematical Sciences, Chalmers University of Technology, Gothenburg, Sweden; University of Minnesota

**Keywords:** antibiotic resistance, bacterial genomes, environment, insertion sequences, metagenomics, resistome

## Abstract

Insertion sequences (ISs) are abundant mobile genetic elements on bacterial genomes, responsible for mobilization of many genes, including antibiotic resistance genes (ARGs). As ARGs often occur in similar genetic contexts, understanding which ISs tend to be associated with known ARGs could be a first step toward discovering novel ARGs through predictive or experimental strategies. This could be valuable, as early identification of ARGs in pathogens could facilitate surveillance, confinement actions, molecular diagnostics, and drug development. Here, we present a comprehensive analysis of the association of specific ISs with known ARGs. A large collection of bacterial genomes was used to characterize the immediate context of 2,437 known ARGs and 3,768 ISs. While many ARGs were consistently found close to specific ISs, the contexts around all ISs were more variable. Nevertheless, a subset of individual ISs, as well as tentative composite transposons, showed significant associations with ARGs. These included, e.g., insertion sequences classified as IS*6*, Tn*3*, IS*4*, and IS*1* that were not only strongly associated with diverse ARGs but also highly abundant in pathogens. Therefore, we conclude that the context of this subset of ISs and tentative composite transposons would be particularly valuable to discover novel ARGs through modeling or empirical approaches. A set of 1,891 metagenomes were analyzed to identify environments where those ISs commonly associated with ARGs were particularly abundant. The associations found in metagenomes were similar to those found in genomes.

**IMPORTANCE** The emergence and spread of antibiotic resistance genes (ARGs) among pathogens threaten the prevention and treatment of bacterial infections as well as our food production chains. Early knowledge about mobile ARGs that are present in pathogens or that have the potential to become clinically relevant could help mitigate potential negative consequences. Recently, exploring integron gene cassettes was shown to be successful for identifying novel mobilized ARGs, some of which were already circulating in pathogens. Still, only a subset of ARGs is mobilized by integrons, and the contexts of other mobile genetic elements associated with ARGs remain unexplored. This includes insertion sequences (ISs) responsible for the mobilization of many ARGs. Our analyses identified ISs, species, and environments where ARG-IS relationships are particularly strong. This could be a first step to guide the discovery of novel ARGs, while also providing insights into mechanisms involved in the mobilization and transfer of ARGs.

## Abstract (precis)

The emergence and spread of antibiotic resistance genes (ARGs) among pathogens threaten the prevention and treatment of bacterial infections as well as our food production chains. Early knowledge about mobile ARGs that are present in pathogens or that have the potential to become clinically relevant could help mitigate potential negative consequences. Recently, exploring integron gene cassettes was shown to be successful for identifying novel mobilized ARGs, some of which were already circulating in pathogens. Still, only a subset of ARGs is mobilized by integrons, and the contexts of other mobile genetic elements associated with ARGs remain unexplored. This includes insertion sequences (ISs) responsible for the mobilization of many ARGs. Our analyses identified ISs, species, and environments where ARG-IS relationships are particularly strong. This could be a first step to guide the discovery of novel ARGs, while also providing insights into mechanisms involved in the mobilization and transfer of ARGs.

## INTRODUCTION

Antibiotic resistance genes (ARGs) gradually accumulate and spread among human pathogens, eventually contributing to increased morbidity and mortality and to social costs (1, 2). While mutations in preexisting genes are a common cause of antibiotic resistance, horizontal gene transfers are posing an even greater challenge by being the main mediators of ARGs among versatile bacterial strains and species (3).

Identifying new ARGs before they become widespread can be valuable for many reasons; awareness of such ARGs may, for example, provide the means for early confinement actions in order to reduce further spread. By restricting exposure to antibiotics in the environments that contain such ARGs, we could reduce the selective forces that maintain ARGs in bacterial communities and subsequently diminish risks for their further transfer to human pathogens. Knowledge of their sequences and the phenotypes that they provide may also facilitate improved molecular diagnostics and thus treatment of bacterial infections. Finally, an understanding of the wider set of resistance mechanisms that could threaten the use of novel therapeutic agents could be considered already during the design phase of new drugs.

New ARGs have been identified both through functional screening of bacterial communities (functional metagenomics) (4, 5) and through modeling, where structural similarities to other related ARGs are utilized (6–8). A plausible hypothesis is that novel ARGs to some extent also share other features with known ARGs, including their probability to occur in association with certain mobile genetic elements. In line with this hypothesis, we have employed focused amplicon sequencing techniques to explore gene cassettes accumulated in class 1 integrons from two polluted river sediments in India (9). This approach not only led to the discovery of a novel sulfonamide resistance gene (*sul4*) but also proved the mobile context of *sul4*, which is an important factor for determining the associated risk for spread (10, 11). Applying functional metagenomics on the same gene-cassette amplicons also led to the identification of a completely new aminoglycoside resistance gene (*gar*), which until then had passed unnoticed in clinical isolates (12).

Associations similar to those found between ARGs and class 1 integrons are also expected between ARGs and the transposable elements (TEs) that are commonly responsible for the capture and intracellular mobilization of ARGs and other genes within and between chromosomes and plasmids (13). The prokaryotic TEs, which include unit transposons and insertion sequences (ISs), are DNA segments that could autonomously move and facilitate mobilization of adjacent regions, primarily using a nonreplicative mechanism (13, 14). Unit transposons involve transposase and accessory/passenger genes surrounded by target site duplications (TSDs) and inverted repeat (IR) terminal motifs. In contrast, ISs are comparatively small elements typically comprised of only one transposase gene that is surrounded by the terminal motifs. Insertion sequences could also form composite transposons which involve two flanking ISs, responsible for insertion and excision of large regions of DNA sequences potentially carrying arrays of ARGs and other genes.

Insertion sequences are classified primarily based on their catalytic domains. There are four different catalytic nuclease domains, including DD(E/D), HUH, phosphoserine, and phosphotyrosine site-specific recombinase, each of which could be found in transposases, invertases, or resolvases (15). The most common transposases in sequenced bacterial genomes contain a DDE domain, characterized by three conserved carboxylate residues (Asp, Asp, and Glu) in its active site, responsible for coordinating two metal ions needed for DNA catalysis. ISs with a DDE domain are widely present across bacteria, with substantial variability in the overall protein sequence (16).

The association between ISs and ARGs was recognized earlier (13, 17). Still, however, a comprehensive analysis of the association between ARGs and ISs is lacking and it is currently not fully clear which types of known and novel resistance genes are mediated by the members of this diverse set of mobile genetic elements. The recent and rapid accumulation of bacterial genomic and metagenomic data in public repositories has provided new opportunities for determining such associations.

We hypothesize that knowledge of the genetic contexts around ISs and known ARGs, revealing the details of their association, will enable a better understanding of the molecular mechanisms driving mobilization and the emergence of resistance genes in pathogens (18). In this study, our aim was thus to analyze the context of IS elements in publicly available sequenced bacterial genomes in order to characterize their associations with ARGs. Among the different families of IS elements, we focused on those that contain DDE domains as they are the most abundant and well-described insertion sequences. Moreover, the structure of two ISs surrounding the mobilized DNA segment (i.e., composite transposons) facilitates certain strategies to discover novel ARGs such as functional screening of PCR amplicons (9). Thereby, genetic contexts surrounded by pairs of ISs (providing potential binding sites for primers) were annotated and tentative composite transposons with variable content were described.

## RESULTS AND DISCUSSION

### Genetic context around ARGs and ISs.

Searching for ARGs listed in the ResFinder database against sequenced genomes in the NCBI Genome database resulted in 877,819 hits (see Fig. S1 in the supplemental material). The most common were resistance genes, providing resistance to beta-lactam and aminoglycoside antibiotics, which also had the highest diversity based on the total number of unique genes encountered. Searching the NCBI nonredundant protein database for matches against IS domains resulted in 798,396 hits, which were clustered into 61,941 IS variants (at least 90% protein amino acid identity [see supplemental file 1 at https://figshare.com/s/4d894dd96ab015c79639]). Data representing the abundance of unique IS variants and their frequency on sequenced genomes are presented in Fig. S2.

10.1128/mSphere.00418-20.1 FIG S1 Frequency of ARGs on all genomes separated by the type of antibiotic that they provide resistance to. The line plot shows the frequency of unique ARGs clustered with 95% identity. Download FIG S1, TIF file, 0.2 MB. Copyright © 2020 Razavi et al. 2020 Razavi et al. This content is distributed under the terms of the Creative Commons Attribution 4.0 International license.

10.1128/mSphere.00418-20.2 FIG S2 Frequency of ISs on sequenced genomes separated by different DDE domains. The line plot shows the frequency of unique IS names. Download FIG S2, TIF file, 0.1 MB. Copyright © 2020 Razavi et al. 2020 Razavi et al. This content is distributed under the terms of the Creative Commons Attribution 4.0 International license.

**FIG 1:**
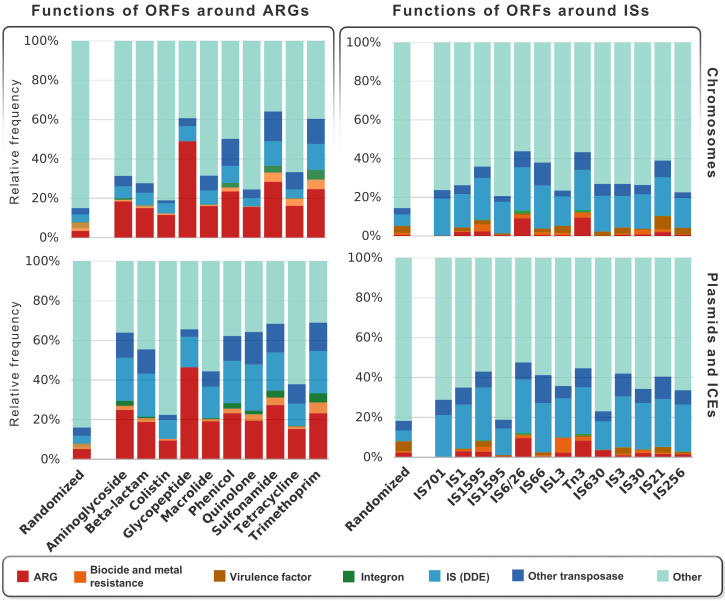
Relative frequencies of putative functions of the open reading frames (ORFs) within 10 ORF distance (see Materials and Methods and Fig. 7) from ARGs (left panel) and IS variants (right panel), in chromosomes and in plasmids and ICE sets, which are grouped by classes of antibiotics and IS domains, respectively. Increasing the ORF distance will inflate the “other” category, probably reflecting an increase in genes not functionally associated with the transposon. We selected an ORF distance of 10, which is roughly where we observed that the inflation of the “other” categories began more clearly.

Analysis of the genetic contexts around the ARGs resulted in a detailed view of the functions of the neighboring genes (Fig. 1, left panel; for more details, see supplemental file 2 at https://figshare.com/s/8a712bff64fb93851a14). Permutation tests showed that the abundance of ARGs around themselves was clearly higher than expected by chance (*P* < 10^−15^), demonstrating that ARGs often cluster together on bacterial genomes. These clusters were not made up of genes that are functionally connected, such as those encoding chromosomal multicomponent efflux pumps, but consisted of mobile, acquired ARGs where a single gene is sufficient for providing the resistance phenotype. An exception was, however, the vancomycin resistance genes, where genes in an operon are functioning together. As these represented only about 1% of the identified ARGs, their presence does not affect our conclusion, especially considering that the *P* value was <10^−15^. Clustering of genes is a selection process that may provide efficient coregulation (i.e., a common promoter in integrons or operons), decreases the likelihood of deleterious mutations, and enhances intra- and intercellular mobility (19).

Gene functions around the ISs are presented in Fig. 1 (right panel; for more details, see supplemental file 3 at https://figshare.com/s/dbc1a9063d98d4195cb0). We found that the ARGs (red bars) were not as abundant around ISs as they were around other ARGs (Fig. 1, left panel). This is not surprising since ISs may insert themselves in many different regions of bacterial genomes, which might result in, in addition to mobilizing ARGs, many other adaptive functionalities, including modulating metabolism and virulence (17).

The relative frequencies of TEs (ISs and other transposases, indicated in blue shading in Fig. 1) around ARGs were higher on plasmids and ICEs (i.e., integrative conjugative elements) than in chromosomes. This is aligned with the role of TEs in capturing and relocating ARGs within bacterial genomes, including the plasmids and ICEs that act as important transmission vectors for ARGs (20).

**FIG 2:**
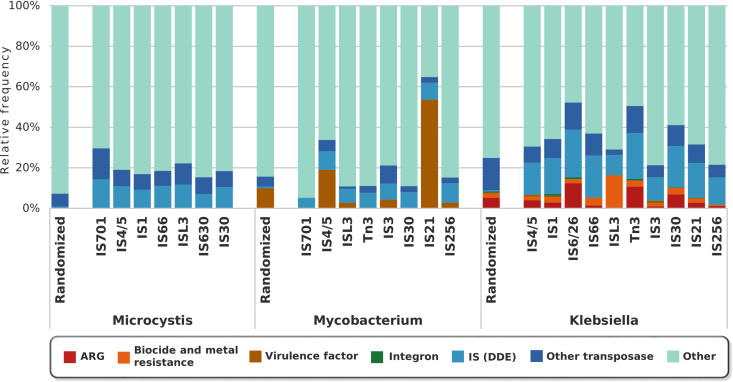
The relative frequencies of putative functions of the ORFs around IS domains in three groups of bacteria are presented. The antibiotic selection pressure in the environment that the bacteria inhabit and the extent of horizontal gene transfer that they engage in appear to be linked to the association of IS variants with ARGs and virulence factors. Only chromosomal ORFs within 10 ORF distance away from each IS were considered in the analyses. As a comparison, the average relative frequencies of the contents within 10 ORF distance of 10,000 randomly selected genes on the chromosomes of the included genomes are presented for each group.

Insertion sequences showed diverse association patterns in bacteria that are abundant in different environments (Fig. 2). In Fig. 2, we have chosen to present bacterial genera that live under quite different conditions. Members of the genus *Microcystis* are often responsible for freshwater blooms and belong to the phylum *Cyanobacteria* (21). They thrive mostly in environments that presumably have low antibiotic selection pressure. As Fig. 2 shows, ISs on sequenced *Microcystis* genomes have no apparent associations with acquired ARGs but are surrounded by transposable elements and probably other genes more critical for the niches they occupy. Members of the genus *Mycobacterium* are Gram-positive bacteria, some of which are pathogenic and could cause tuberculosis in humans and cattle. Some mycobacteria are hence more likely to be exposed to antibiotics than *Microcystis*, but they are known to be less dependent on acquired ARGs due to a variety of intrinsic and mutation-based resistance determinants (22). Indeed, we found that ISs in mycobacteria had a much lower level of association with acquired ARGs, but the ISs were mostly surrounded by virulence factors, transposable elements, and genes with other functions. In contrast, members of the genus *Klebsiella* have stronger associations of ISs with ARGs. This genus includes important nosocomial pathogens that regularly face strong antibiotic selective pressure and that engage frequently in horizontal gene transfer (23). It is likely that persistent exposure to antibiotics has apparently selected for ARGs and/or enhanced association of ISs with ARGs on their genomes.

### Association of ISs with ARGs.

**FIG 3:**
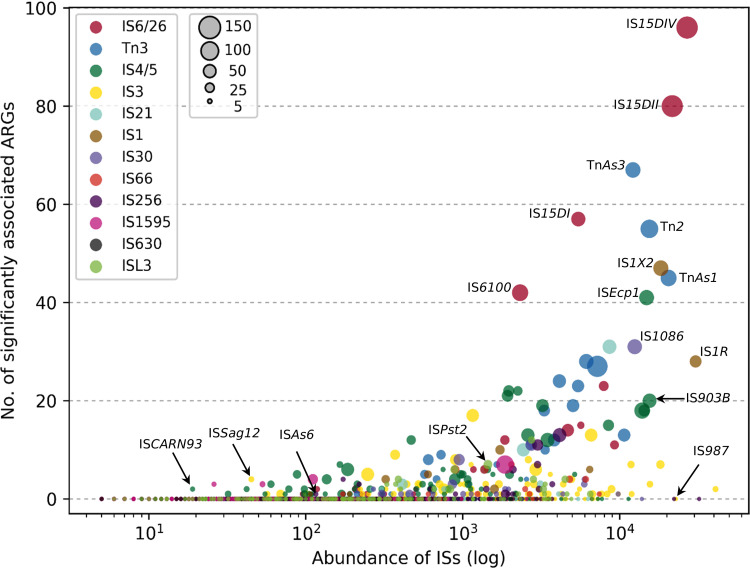
Distribution of ISs that are significantly associated with different types of ARGs within 10 ORF distance (see Materials and Methods). Different colors show various IS domains, and the size of the circles represents the presence of their associations with ARGs in different bacterial genera.

Next, we assessed the pairwise association between ISs and ARGs (see Materials and Methods and supplemental file 4 at https://figshare.com/s/d963080d06e9c5c8a573). All associations were analyzed using a permutation test to differentiate between the statistically significant associations and those that could be explained purely based on chance. The significant associations (*P* < 0.001) of ISs and ARGs are presented in Fig. 3 (for more details, see supplemental file 5 at https://figshare.com/s/cdcdf8533ba1864a34d6).

Insertion sequences with many significant associations with ARGs are represented in the upper right section of Fig. 3. These include members of the IS*6*/*26* domain (IS*15DIV*, IS*15DI*, IS*15DII*, and IS*6100*), the Tn*3* domain (Tn*2*, Tn*As3*, and Tn*As1*), the IS*1* domain (IS*1R* and IS*1X2*), and the IS*4*/*5* domains (IS*903B* and IS*Ecp1*), which are all well known for mobilizing ARGs in different bacterial species (13). Their high abundance and strong associations with ARGs could be a result of their initial presence in pathogens and/or in environments that harbor both gene types. It is also plausible that ISs that had mobilized ARGs in the past are still maintained and continue to accumulate ARGs under conditions of antibiotic selection pressure. This would result in coselection for IS-bearing bacteria, thereby providing opportunities for them to form associations with yet other ARGs. Nevertheless, other factors, such as a higher tendency for self-replication or a lower cost of transposition for the host, could help specific ISs to outcompete others and enhance associations with ARGs.

The lower left corner of Fig. 3 shows less-abundant ISs, where IS*Sag12*, IS*As6*, and IS*CARN93* are examples. These ISs have fewer significant associations with ARGs, probably due to their low abundance in the genomic database, which could result in lower statistical power in permutation tests. The possibility cannot be excluded that there might be bacteria where such ISs are significantly associated with more ARGs, but if so, those genomes remain to be sequenced. Interestingly, there were also more-abundant ISs that showed few significant associations with ARGs, suggesting that they may have other primary roles or taxonomic preferences. This includes some members of the IS*256* family that are known to modulate virulence in Gram-positive bacteria (24), members of the IS*L3* family (e.g., IS*Pst2*) known to modulate metabolic activities, and IS*987* (isoform IS*6110*). The latter is exclusive to the Mycobacterium tuberculosis complex and has even been used as a strain-specific marker for typing in epidemiological studies of tuberculosis (25). Transposition of IS*987* is controlled by the host due to its possible deleterious effect (26). With very slow transposition events, it has a limited chance to be associated with various ARGs.

The ISs with the most significant associations displayed a similar level of diversity in terms of the antibiotic classes to which the associated ARGs provide resistance (Fig. S3a). This suggests that there is no or limited differential preference between ISs and antibiotic classes. This was as expected, in contrast to ISs with few associations, which showed a much larger range of variability of associated ARGs (Fig. S3b). While the associations with integrons and conjugative plasmids seem to be different for these two groups, their associations with other ISs are almost the same (see supplemental file 4 at https://figshare.com/s/d963080d06e9c5c8a573). Insertion sequences such as IS*15DII*, IS*1326*, and Tn*As3* have integrons in more than 70% of their genetic contexts (i.e., within a 10 ORF distance) and they are among the most abundant ISs on plasmids. In contrast, ISs with few significant associations with ARGs, such as IS*Ecp38*, IS*Sau4*, and IS*Sag10*, showed very weak associations with integrons and plasmids. We cannot, however, rule out the possibility that the members of the latter group might have associations with integrons and plasmids in bacterial genomes that have yet to be sequenced. Integrons offer rapid bacterial adaptation in response to antibiotic stress by capturing and expressing ARGs in the form of gene cassettes. By accessing versatile resistance gene cassettes, ISs involved in the mobilization of integrons would thus have a higher chance of being coselected and maintained on bacterial genomes under selection pressure from antibiotics.

10.1128/mSphere.00418-20.3 FIG S3 Significant association of selected IS names with different ARG families within a distance corresponding to 10 ORF distance. (a) ISs with many significant associations with ARGs. (b) ISs with fewer significant associations with ARGs. The selected insertion sequences have abundance values of greater than 1,000 in genome data. Download FIG S3, TIF file, 0.1 MB. Copyright © 2020 Razavi et al. 2020 Razavi et al. This content is distributed under the terms of the Creative Commons Attribution 4.0 International license.

### Identifying tentative composite transposons.

We used the Jaccard index to assess the variability of tentative composite transposons, where a low value indicates a higher level of variability of genetic contents between pairs of ISs (see supplemental file 6 at https://figshare.com/s/b5154ea3633ccfa9265d). For instance, tentative composite transposons of IS*66* (IS*Ec49*-IS*682*) had a high average Jaccard index (>0.95), showing that their genetic content is relatively static. In contrast, the tentative composite transposons of IS*26* (IS*15DIV*-IS*15DIV*) had a low average Jaccard index (<0.1), showing a high level of variability with few shared genes.

**FIG 4:**
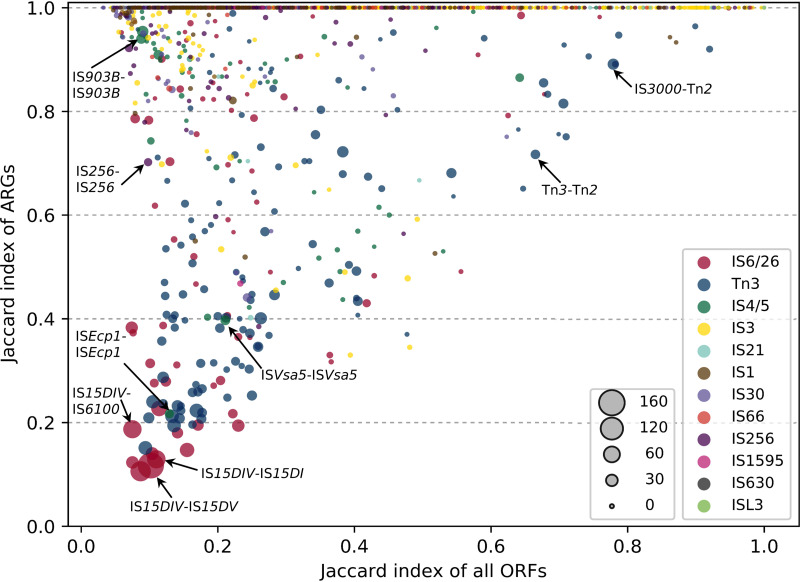
The variability of all ORFs versus all ARGs within tentative composite transposons. The colors represent different IS domains, and the sizes of the markers are scaled to reflect the ARG richness within tentative composite transposons.

The variability of all the ORFs in relation to the variability of the identified ARGs within tentative composite transposons was therefore analyzed (Fig. 4). Tentative composite transposons with higher ARG richness (bigger marker size) had, in general, lower Jaccard indices. This shows that the dynamic transposition of ISs is connected to high ARG richness such that different instances of the tentative composite transposons should mobilize different genes, including ARGs, in order to be possibly coselected and maintained with ARGs in the genomes of various bacterial hosts. This is further illustrated in Fig. S4, where ISs with higher variability of ARGs are shown to be generally more abundant. There were, however, some tentative composite transposons belonging to the Tn*3* family (e.g., IS*3000*-Tn*2*, Tn*3*-Tn*2*) that, despite their low variability (i.e., high Jaccard index), had relatively high ARG richness. Since some of the members of the Tn*3* family are unit transposons (13), they could mobilize genetic materials and enhance their associations with ARGs without being dependent on another IS. Transposition of the second IS within the unit transposons might have been happened once and have been maintained since then. Thus, despite the high level of ARG richness between the two ISs, the variability of the surrounded context is low. The variability seen between two unit transposons (e.g., Tn*3*-Tn*2*), or between those ISs that move via rolling-circle transpositions (e.g., IS*Ecp1*), in close proximity to each other might not be as meaningful as that associated with a pair of ISs that form a composite transposon. However, the Jaccard index could rather represent the variable genetic context captured by each of the transposases in these groups alone. Nevertheless, analyses of variability within composite transposons along with other pieces of information about ISs could help identify dynamic ISs with strong associations with ARGs.

10.1128/mSphere.00418-20.4 FIG S4 The variability of all ARGs within tentative composite transposons versus the abundance of tentative composite transposons. The colors represent different IS domains, and the sizes of markers are scaled to reflect the ARG richness within tentative composite transposons. Download FIG S4, TIF file, 0.1 MB. Copyright © 2020 Razavi et al. 2020 Razavi et al. This content is distributed under the terms of the Creative Commons Attribution 4.0 International license.

### Insertion sequences in metagenomes.

**FIG 5:**
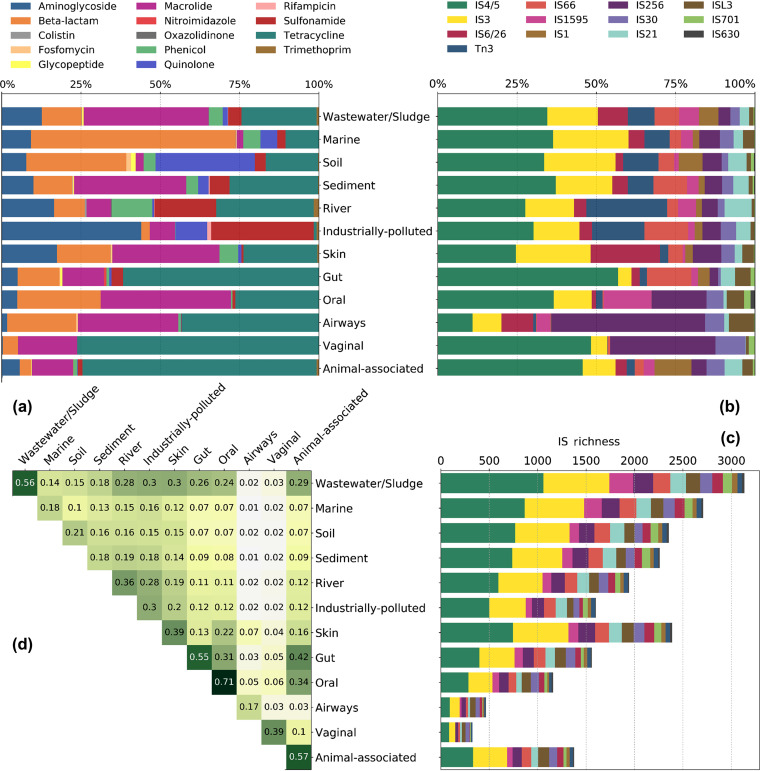
Insertion sequences and ARGs in metagenomic data sets. (a) Relative abundances of ARGs categorized by ARG families. (b) Relative abundances of IS names categorized by IS domains. (c) Number of different ISs (IS richness) found in each environment grouped by IS domains. (d) Bray-Curtis measurement matrix of environments. The data show similarities between different environments over the presence/absence data of ISs. The main diagonal shows the within-environment similarities.

Next, we investigated the abundance of ISs and ARGs in a large collection of metagenomes representing human and animal microbiomes as well as the external environment (Fig. 5a and b) (see supplemental file 7 at https://figshare.com/s/3f0395f4c8ba0f55d91e and supplemental file 8 at https://figshare.com/s/07f36661d118d4ef3404). The IS richness data, calculated from the unique number of identified IS names, are presented in Fig. 5c. The metagenomes from external environments (e.g., wastewater/sludge and marine) had the highest IS richness while the human-related metagenomes (e.g., oral, airway, and vaginal) had the lowest, probably in part reflecting the level of taxonomic diversity (27). All IS domains were present in all environments, but wastewater/sludge contained the largest collection of different ISs in each domain. Insertion sequences belonging to IS*4*/*5* and IS*3* domains were dominant in most of the environments in terms of abundance (Fig. 5b) and richness (Fig. 5c). However, some of the IS domains with lower IS richness (e.g., IS*256*, IS*6*/*26*, and Tn*3*) were found to have considerably increased relative abundances in certain environments (Fig. 5b). This highlights their possible associations with specific bacteria common in these environments or a selection under the given environmental conditions. For instance, members of IS*6*/*26* domain, including IS*257R1*, IS*257R2*, IS*431R*, and IS*431mec*, were among the most abundant ISs in the skin metagenomes. From the analysis of the bacterial genomes, we also found that these ISs were common in Staphylococcus aureus, which is a commensal that is highly abundant in the human microbiome, especially on the skin. A similar pattern could be found in the vaginal metagenomes, where high abundances of ISs within the IS*256* (i.e., IS*1201* and IS*Ldl2*) and IS*30* (i.e., IS*Ldl3*) domains were observed. These ISs are often encountered in *Lactobacillus*, which is common in the vaginal microflora. Moreover, association of Tn*3* (e.g., Tn*5393*, Tn*As3*, Tn*As2*, and Tn*As1*) and IS*6*/*26* (e.g., IS*15DIV* and IS*6100*) with ARGs (see supplemental file 5 at https://figshare.com/s/cdcdf8533ba1864a34d6) might explain their increased abundance in industrially polluted environments. From the genomic analyses, we found that these ISs have strong associations with ARGs and, possibly through these associations, the genetic contexts containing both ISs and ARGs were selected by the high levels of antibiotics found in those environments (28). Moreover, the distributions of ISs were compared between environments (Fig. 5d; see also Materials and Methods). This analysis showed that some environments were rather similar to each other in terms of IS composition. For instance, the human gut- and animal-associated metagenomes were highly similar, especially those associated with the oral and gut microbiomes. Moreover, wastewater/sludge metagenomes were found to share ISs with most of the other environments to a large degree, possibly because it is the most diverse environment studied (with respect to ISs).

To further investigate the variability between individual metagenomic data sets, we performed a multidimensional scaling (MDS) analysis based on the IS abundance (Fig. S5). This showed that most of the human- and animal-associated metagenomes (e.g., oral, animal-associated, and gut metagenomes) were separated from external environments (e.g., marine, soil, and river) whereas wastewater/sludge metagenomes are mostly found between them. Furthermore, the animal and human gut metagenomes showed a lower level of within-environment variability than the metagenomes from external environments, such as the marine, soil, and sediment metagenomes, which were more diverse. This is also aligned with the values shown on the main diagonal of Fig. 5d, in which it is demonstrated that environments with higher within-environment similarities also have higher cohesion, as shown in Fig. S5. It could very well be that human/animal gut and wastewater/sludge environments are less variable and thus that the composition of ISs are more similar across those metagenomes, whereas external environments contain many more and variable niches, and thereby taxonomic diversity, which in turn influences the composition of ISs.

10.1128/mSphere.00418-20.5 FIG S5 Similarity of different metagenomic datasets described by the abundances of ISs. More-similar metagenomes in terms of Bray-Curtis measures are closer to each other. Some human- and animal-associated and external environments are relatively well separated from each other. Three clusters of metagenomes representing different environmental conditions are marked as follows: (a) deep-sea hydrothermal vent of Mid-Cayman Rise; (b) marine sediments of estuaries in Australia; (c) collection of presumably polluted metagenomes from China (MG-RAST: MGP90129), India (N. P. Marathe, C. Pal, S. S. Gaikwad, V. Jonsson, E. Kristiansson, and D. G. J. Larsson, Water Research 124:388-397, 2017, https://doi.org/10.1016/j.watres.2017.07.060), and Africa (MG-RAST: MGP87146). Download FIG S5, TIF file, 0.3 MB. Copyright © 2020 Razavi et al. 2020 Razavi et al. This content is distributed under the terms of the Creative Commons Attribution 4.0 International license.

### Associations of ISs with ARGs in metagenomes reflect those found in genomes.

**FIG 6:**
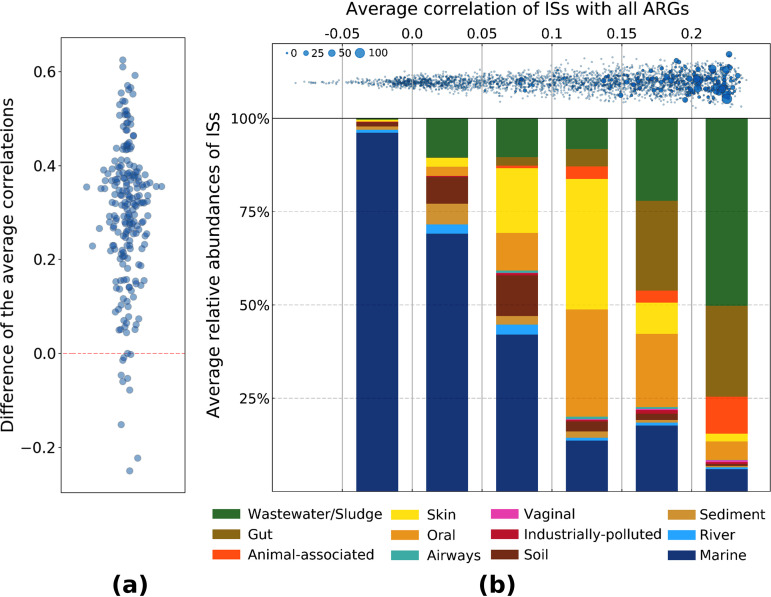
Tracing the associations of ISs with ARGs, identified from analyzing bacterial genome data, in metagenomes. (a) A scatterplot showing the difference between average correlations of the relative abundances of ISs with two groups of ARGs across different metagenomes, i.e., those ARGs significantly associated with ISs from the genome data and those less extensively associated with ISs (see Materials and Methods). A Wilcoxon test confirms a highly significant difference between them (absence of a difference is indicated by a horizontal dashed red line). (b) (Top panel) Average Spearman correlation of ISs with all the ARGs. The sizes of the markers are scaled according to the ARG richness from genome data. (Bottom panel) Stack bar chart showing the distribution of ISs across different environments. The average of relative abundances of ISs across metagenomes in each environment was used.

Most of the ISs with significant associations with ARGs correlated better with their respective ARGs than with other ARGs across different metagenomes (Fig. 6a). The Wilcoxon test showed that the average correlations of ISs with these two groups of ARGs were significantly different (*P* < 10^−15^). This demonstrates that the specific associations observed in the bacterial genome data are also present in bacterial communities.

Insertion sequences with a wide range of correlational spectra are abundant in different environments (Fig. 6b). In presumably somewhat less human-impacted environments such as marine environments and soil, ISs with weaker correlations with ARGs are abundant, whereas those with stronger correlations are found mostly in human-, animal-, and wastewater-impacted environments. Genomic analyses could provide taxonomical clues on distribution of ISs in different environments. For instance, strong associations of IS*Atsp1* (average correlation, 0.03) with *Cyanobacteria* phylum, IS*431mec*/*R* (average correlation, >0.07) with S*taphylococcus* genus, and IS*26*, including IS*15DIV* and IS*15DII* (average correlation, >0.2), with several enteric bacteria were found to be aligned with increased abundances of ISs in marine, skin, and wastewater-impacted metagenomes, respectively.

There are ISs with a high average correlation with ARGs in metagenome data, even though no significant associations have been found in the genome data. Metagenomic data sets represent complex microbial communities, allowing ISs that are located on the same bacterial genomes or in the concomitant hosts to follow the same pattern. For instance, IS*Ec23* (average correlation, 0.22) has been found to be abundant in genera *Escherichia*, *Salmonella*, and *Klebsiella* (more than 95% of 2,502 occurrences in genome data). These bacterial genera also contain ARGs on their genomes, but probably not in the regions surrounding IS*Ec23*. Moreover, IS*Lpl1* (average correlation, 0.22) is abundant in Lactobacillus plantarum (85% of 1,845 occurrences), which is a probiotic bacterium found in food and human gastrointestinal tract (29). A metagenomic data set from gut- or wastewater-impacted environments probably contains both IS*Lpl1* and ARG-associated ISs, causing increased correlation. Nevertheless, we must also consider that metagenomic samples might contain novel associations of ISs with ARGs, or even different bacterial hosts containing new associations that have not been identified through the whole-genome sequencing approach.

### ISs with increased potential to harbor novel ARGs.

Considering the genomic and metagenomic analyses of ISs, we would prioritize exploring the content around the groups of ISs listed in Table 1. ARGs are more often associated with TEs (see Fig. 1, left panel) than with, for instance, class 1 integrons, as most ARGs are not present as gene cassettes. Exploring the content around specific ISs could hence be critical for identifying many as-yet-unrecognized ARGs. The first group contains ISs with both strong associations with ARGs and variable genetic contexts. They are all abundant in pathogens that thrive in human/animal-associated and wastewater-impacted environments.

**TABLE 1:** ISs associated with a high diversity of ARGs (group 1) or with a generally variable context (group 2)

Group	Family	IS name	Abundance (on genomes)	Jaccard index^*a*^	ARG richness value	Selected host(s) (genus)	Environment(s)^*b*^
1	IS*6*	IS*15DIV*	26,941	0.086	113	*Salmonella*, *Klebsiella*, *Escherichia*; *Pseudomonas*, Acinetobacter, *Enterobacter*	W, G, A
		IS*6100*	2,323	0.297	43	*Salmonella*, *Klebsiella*, *Escherichia*; *Pseudomonas*, Acinetobacter, *Enterobacter*	W, I, R
		IS*431*	9,286	0.214	17	*Staphylococcus*, *Enterococcus*, *Streptococcus*	S, A
		IS*1216E*	7,930	0.030	20	*Enterococcus*; *Staphylococcus*; *Streptococcus*	W, A, O
	Tn*3*	Tn*5393*	7,214	0.26	31	*Salmonella*, *Escherichia*, *Klebsiella*, *Pseudomonas*, *Enterobacter*, *Sphingobium*	O, I, W
		Tn*As1*	20,532	0.176	54	*Salmonella*, *Escherichia*, *Klebsiella*, *Pseudomonas*, *Shigella*, *Enterobacter*	W, A, G
		Tn*2*	15,492	0.165	64	*Salmonella*, *Klebsiella*, *Escherichia*, *Shigella*, *Enterobacter*	O, A, W
	IS*1380*	IS*Ecp1*	14,872	0.185	44	*Enterococcus*; *Escherichia*; *Salmonella*; *Enterobacter*; *Klebsiella*; *Citrobacter*	W, Se, A
	IS*5*	IS*903B*	15,540	0.052	44	*Escherichia*; *Salmonella*; *Enterobacter*; *Klebsiella*; *Citrobacter*	W, G, So
		IS*5D*	8,502	0.08	39	*Klebsiella*, *Escherichia*, *Enterobacter*, *Salmonella*, *Citrobacter*	W, G, A
	IS*256*	IS*406*	3,006	0.263	24	*Escherichia*; *Salmonella*; *Polaromonas*	W, Se, A
		IS*1542*	223	0.067	13	*Enterococcus*; *Bacillus*; *Lactobacillus*; *Jeotgalibaca*; *Aerococcus*; *Listeria*	W, A, O
		IS*256*	4,146	0.098	15	*Staphylococcus*, *Enterococcus*, *Clostridioides*, *Streptococcus*	O, W
	IS*21*	IS*1326*	8,627	0.26	27	*Salmonella*, *Escherichia*, *Klebsiella*, *Pseudomonas*, *Enterobacter*	W, R, Se
	IS*30*	IS*Apl1*	950	0.269	8	*Escherichia*, *Lactobacillus*, *Mannheimia*, *Salmonella*, *Histophilus*	O, G, A

2	IS*256*	IS*Xax1*	1,474	0.084	0	*Xanthomonas*, *Methylomonas*, *Legionella*, *Rickettsia*, *Desulfuromonas*	W, R, Se
	IS*5*	IS*1021*	536	0.087	0	*Ralstonia*, *Variovorax*, *Halomonas*, *Burkholderia*	A, W, Se
	IS*3*	IS*Psa2*	831	0.078	0	*Piscirickettsia*	M
	IS*982*	IS*195*	693	0.060	0	*Prevotella*, *Bacteroides*, *Flavobacterium*	O, G, A

a For unit transposases such as members of Tn*3* families, averages over all the identified composite transposons with at least one Tn*3* transposase were used.

b M, marine; W, wastewater/sludge; G, gut; So, soil; S, skin; A, animal associated; R, river; O, oral; Se, sediment; V, vaginal; P, industrially polluted.

We should also consider the emergence of novel ARGs through other ISs that might be less extensively associated with known ARGs. Table 1 lists some ISs that constitute parts of highly variable tentative composite transposons, which thereby have the potential to also carry novel resistance determinants. For instance, IS*Xax1* and IS*1021* are mostly abundant in the genera *Xanthomonas* and *Ralstonia*, respectively. We have not found any association of IS*Xax1* and IS*1021* with the known ARGs, but exposure to antibiotics could create selection pressure that could prompt such ISs to mobilize known and novel ARGs. Similarly, IS*psa2* in fish pathogens or IS*195* in members of oral, vaginal, and gut microbiota could impose such risks as well.

With a well-designed strategy, the contexts of ISs already associated with a high diversity of known ARGs may be explored further to find novel putative ARGs. Knowledge of such associations can be used to guide the analysis of whole-genome sequencing data to find ORFs around ISs of interests with regard to candidate genes that could confer resistance to antibiotics. Moreover, targeted amplicon sequencing may be employed to extract genetic context within composite transposons of interest from complex bacterial communities. In such a culture-independent approach, the recovered genetic material could be amplified by specific primers that could target pairs of ISs, which could then be sequenced, preferably with long-sequencing technologies (e.g., SMRT or Nanopore), to explore the captured ORFs. Functional metagenomic techniques used on amplified composite transposons may also be applied to increase the chances of finding completely novel classes of ARGs. Moreover, there is a possibility of using inverse PCR to explore contexts around certain ISs.

### Conclusions.

We have described the associations between known ARGs and ISs containing common DDE domains among bacteria. ISs with strong associations with ARGs were identified, and tentative composite transposons with the potential of mobilizing novel putative ARGs into human pathogens were suggested. With a well-designed strategy, the contexts of these ISs along with the contexts around ARGs may be explored further to find novel putative ARGs. This could involve functional or sequence-based screening of ORFs around specific genes, and particularly around those located within certain composite transposons. This report also provides a general framework to explore other mobile genetic elements such as ISCR and unit transposons as well as unknown insertion sequences to facilitate discovery of mobile novel ARGs.

## MATERIALS AND METHODS

### Exploring genetic contexts around ARGs.

ARGs from the ResFinder database (downloaded 15 April 2018) (30) were used as it contains only mobile genes. Genes were first translated using Prodigal (v2.6.3) (31) and used to identify ARGs in the NCBI Genome database, which is the repository of bacterial sequenced genomes (downloaded 15 August 2019; contains 333,456 genomes). The genomes were annotated as follows. Prodigal (v2.6.3) was used to detect open reading frames (ORFs); Diamond (v0.9.24.125) (32) was used to search for matches (i.e., with 90% identity and 50% coverage to balance false positives and false negatives for different groups of genes, especially ARGs [33]) against the NCBI nonredundant protein database (downloaded 18 July 2019) and ResFinder, Bacmet (confirmed resistance genes v2.0) (34), and VFDB (downloaded 6 February 2018) were used to specifically annotate ARGs, metal and biocide resistance genes, and virulence factors, respectively. By the use of Mummer3 (35), plasmids from the NCBI database (downloaded 13 July 2018) and ICEs from the ICEberg (v2.0) database (36) were detected in the sequenced genomes.

### Exploring genetic contexts around ISs.

The list of IS families containing DDE domains was identified from the literature (16) and by using hidden Markov model (HMM) protein models from the PFAM, COG, and Conserved Domain databases (see Table S1 in the supplemental material). For detecting members of IS*630*, we used their specific DNA binding domains in the PFAM database (37). Moreover, we included all the transposases from the Tn*3* family in our analyses, even though some of its members function as unit transposons. The models were searched against the NCBI nonredundant protein database using Hmmer3.0 software (38). Proteins belonging to the bacteria with an E value of less than 10^−5^ were clustered by CD-HIT using a 90% identity threshold (39). To find the IS names and families, the unique ORFs containing DDE domains were searched against the ISFinder database (40) using the online BLAST tool available at https://isfinder.biotoul.fr/blast.php (last accessed September 2019) with 50% identity and 50% coverage and also the NCBI nonredundant protein database using Diamond with 90% identity and 50% coverage. The genomes containing matching ORFs were analyzed with the pipeline described above. Throughout the text, we use the following insertion sequence nomenclature. The identified nonredundant ORFs in the sequenced genomes are called “IS variants.” They have been grouped by the “IS names” that were retrieved from the ISfinder database. The IS names are, in turn, structured into IS families according to the ISfinder database. Finally, we use the term “IS domain” to denote IS families that share the same DDE domains as defined by the HMM protein models. We refer to IS names as ISs for simplicity, except in cases in which we explicitly mention the variant, family, or domain to refer to individual ORFs, the assigned family, or the set of ISs with the same DDE domains, respectively.

10.1128/mSphere.00418-20.6 TABLE S1 Protein domains used to collect different IS elements. Download Table S1, DOCX file, 0.01 MB. Copyright © 2020 Razavi et al. 2020 Razavi et al. This content is distributed under the terms of the Creative Commons Attribution 4.0 International license.

### ORF distance metric and relative frequencies.

**FIG 7:**
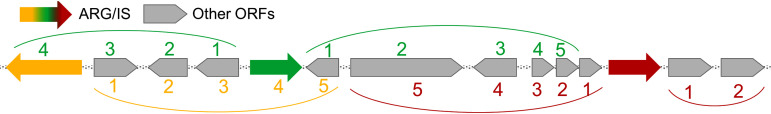
Conceptual example of how ORFs located downstream and upstream of three core genes (each an IS or ARG) were numbered. Note that for each occurrence of an ARG or IS, a new region with surrounding ORFs was analyzed.

Genes around identified ARGs and IS variants were enumerated to determine an estimated distance to neighboring ORFs. We assigned a value of zero to the identified ARG or IS variants and numbered the ORFs in upstream and downstream locations based on their position (Fig. 7). For a given distance, the relative frequencies of gene functions around each ARG or IS variant were calculated by dividing the number of occurrences by the total number of matches of the ARG or IS variants among the analyzed genomes.

### Tentative composite transposons.

Insertion sequences from the same IS domain with an ORF-distance of less than 30 were considered tentative composite transposons. Acknowledging the difficulty of accurately identifying functional composite transposons, we hypothesized that it is more likely that IS pairs with the same protein domain could detect the necessary motifs required for transposition and thus function as composite transposons. Moreover, the lengths of operational composite transposons can differ between IS families and might span more than 30 ORFs. However, the longer the distance used to define tentative composite transposons, the larger the probability of encountering IS copies that were not functionally interacting with each other. With this approach, we could evaluate tentative composite transposons in the sequenced genome data, their various genetic contexts, associations with ARGs, and occurrences in human pathogens.

The ORFs within all the instances of identified, tentative composite transposons were clustered with 90% similarity threshold using CD-HIT. The similarity of the gene contents between two tentative composite transposons was calculated using the pairwise Jaccard index. The Jaccard index of two instances of a composite transposon is defined as the ratio of the number of common genes to the total number of unique genes surrounded by ISs. The mean overall pairwise Jaccard indices were then reported as variability metrics for each composite transposase. Moreover, specific Jaccard indices for ARGs, ISs, and virulence factors were calculated by considering only ORFs from these groups.

### Statistical analyses.

To measure the statistical significance of the associations of ISs and ARGs, permutation tests were employed as follows. All ORFs within the respective genomes (e.g., those containing both ISs and ARGs) were randomly permutated, and for each permutation, the association of interest was calculated. This was repeated 10,000 times, resulting in an empirical null distribution. A normal distribution was fitted to the distribution (using the SciPy package [41]), and the significance, in the form of a one-sided *P* value, was reported, after calculating the probability for a more extreme observation.

### Metagenomic analyses.

In total, 1,891 metagenomic data sets produced by a similar platform, i.e., Illumina sequencing, were selected for the analyses of unique (90% identity threshold) IS variants (Table S2). We selected metagenomes from various environments representing different geographical and environmental conditions from the MGnify (42) and MG-RAST (43) databases. The metagenomes were assigned to groups based on their source as follows. Human microbiomes were divided into gut, vaginal, skin, oral, and airway groups. Animal-associated metagenomes were mostly comprised of animal feces collected from pigs and poultry. External environments were divided into soil, river (water samples from rivers, lakes, and groundwater), marine (water samples from marine environments), wastewater/sludge (samples from different parts of the wastewater cycle, including influents, effluents, active sludge, etc.), and sediment (freshwater and marine sediments) environments. Sediment samples collected in areas strongly impacted by discharges from antibiotic manufacturing were classified as a separate group, referred to as “industrially polluted” (44).

10.1128/mSphere.00418-20.7 TABLE S2 Collection of metagenomic datasets used in this study. Download Table S2, DOCX file, 0.01 MB. Copyright © 2020 Razavi et al. 2020 Razavi et al. This content is distributed under the terms of the Creative Commons Attribution 4.0 International license.

Diamond was used to map the reads to IS variants using an identity threshold of 95% to balance specificity and sensitivity (33). Reads that mapped to several reference proteins were counted multiple times. The relative abundances of IS names, families, and domains were calculated on the basis of the average relative abundances of the corresponding IS variants. The abundances of ARGs were calculated with the same pipeline after clustering them using CD-HIT with an identity threshold of 90%. The relative abundances of ISs and ARGs were reported per million reads.

IS richness and between-sample similarity values were calculated based on downsampled data sets where one million reads were randomly selected without replacement. Metagenomes with fewer than one million reads were removed from this part of the analysis (they included 1 animal-associated, 23 marine, 43 river, 1 sediment, 8 soil, and 34 wastewater/sludge data sets). The similarity of different environments was calculated based on the presence/absence of ISs (i.e., IS names) using the Bray-Curtis similarity measure. The pairwise similarities of metagenomes within and between environments were calculated, and then the average was reported.

Multidimensional scaling (MDS) of all the downsampled metagenomes was performed as follows. Each metagenome was described by the relative abundances of 3,768 ISs and the pairwise dissimilarities between them were calculated according to the Bray-Curtis dissimilarity measure. By using the SMACOF algorithm in the Scikit-learn package (n_components = 2, n_init = 100, max_iter = 1000), metagenomes were visualized in a two-dimensional Cartesian coordinate system (45).

Correlations between relative abundances of ISs and ARGs were calculated as follows. First, for each IS, the significant associations (*P* < 10^−15^) with ARGs were identified from bacterial genome data (i.e., the NCBI Genome database). Then, the average pairwise Spearman correlations of the relative abundances over all metagenomes were calculated. To assess the significance of the correlation values, we also calculated the Spearman correlations of associations of ISs with ARGs not observed in the NCBI Genome database. Then, by using the Wilcoxon test, the significance of the differences in correlations between these groups was assessed.

## References

[1] Tacconelli E, Magrini N, Kahlmeter G, Singh N. 2017. Global priority list of antibiotic-resistant bacteria to guide research, discovery, and development of new antibiotics. World Health Organization, Geneva, Switzerland. https://www.who.int/medicines/publications/global-priority-list-antibiotic-resistant-bacteria/en/.

[2] O’Neill J (ed). 2014. Antimicrobial resistance: tackling a crisis for the health and wealth of nations. https://amr-review.org/sites/default/files/AMR%20Review%20Paper%20-%20Tackling%20a%20crisis%20for%20the%20health%20and%20wealth%20of%20nations_1.pdf. Accessed August 2020.

[3] Woodford N, Ellington MJ. 2007. The emergence of antibiotic resistance by mutation. Clin Microbiol Infect 13:5–18. doi:10.1111/j.1469-0691.2006.01492.x. 17184282

[4] Allen HK, Moe LA, Rodbumrer J, Gaarder A, Handelsman J. 2009. Functional metagenomics reveals diverse β-lactamases in a remote Alaskan soil. ISME J 3:243–251. doi:10.1038/ismej.2008.86. 18843302

[5] Marathe NP, Janzon A, Kotsakis SD, Flach C-F, Razavi M, Berglund F, Kristiansson E, Larsson DJ. 2018. Functional metagenomics reveals a novel carbapenem-hydrolyzing mobile beta-lactamase from Indian river sediments contaminated with antibiotic production waste. Environ Int 112:279–286. doi:10.1016/j.envint.2017.12.036. 29316517

[6] Berglund F, Marathe NP, Österlund T, Bengtsson-Palme J, Kotsakis S, Flach C-F, Larsson DJ, Kristiansson E. 2017. Identification of 76 novel B1 metallo-β-lactamases through large-scale screening of genomic and metagenomic data. Microbiome 5:134. doi:10.1186/s40168-017-0353-8. 29020980 PMC5637372

[7] Lakin SM, Kuhnle A, Alipanahi B, Noyes NR, Dean C, Muggli M, Raymond R, Abdo Z, Prosperi M, Belk KE, Morley PS, Boucher C. 2019. Hierarchical hidden Markov models enable accurate and diverse detection of antimicrobial resistance sequences. Commun Biol 2:294. doi:10.1038/s42003-019-0545-9. 31396574 PMC6684577

[8] Berglund F, Österlund T, Boulund F, Marathe NP, Larsson DJ, Kristiansson E. 2019. Identification and reconstruction of novel antibiotic resistance genes from metagenomes. Microbiome 7:52. doi:10.1186/s40168-019-0670-1. 30935407 PMC6444489

[9] Razavi M, Marathe NP, Gillings MR, Flach C-F, Kristiansson E, Joakim Larsson DG. 2017. Discovery of the fourth mobile sulfonamide resistance gene. Microbiome 5:160. doi:10.1186/s40168-017-0379-y. 29246178 PMC5732528

[10] Martínez JL, Coque TM, Baquero F. 2015. What is a resistance gene? Ranking risk in resistomes. Nat Rev Microbiol 13:116–123. doi:10.1038/nrmicro3399. 25534811

[11] Bengtsson-Palme J, Larsson DGJ. 2015. Antibiotic resistance genes in the environment: prioritizing risks. Nat Rev Microbiol 13:396. doi:10.1038/nrmicro3399-c1. 25915637

[12] Böhm M-E, Razavi M, Marathe NP, Flach C-F, Joakim Larsson DG. 2020. Discovery of a novel integron-borne aminoglycoside resistance gene present in clinical pathogens by screening environmental bacterial communities. Microbiome 8:41. doi:10.1186/s40168-020-00814-z. 32197644 PMC7085159

[13] Partridge SR, Kwong SM, Firth N, Jensen SO. 2018. Mobile genetic elements associated with antimicrobial resistance. Clin Microbiol Rev 31:e00088-17. doi:10.1128/CMR.00088-17. 30068738 PMC6148190

[14] Siguier P, Gourbeyre E, Varani A, Ton-Hoang B, Chandler M. 2015. Everyman’s guide to bacterial insertion sequences. Microbiol Spectr 3:MDNA3-0030-2014. doi:10.1128/microbiolspec.MDNA3-0030-2014. 26104715

[15] Hickman AB, Dyda F. 2015. Mechanisms of DNA transposition. Microbiol Spectr 3:MDNA3-0034-2014. doi:10.1128/microbiolspec.MDNA3-0034-2014. PMC7422641 26104718

[16] Siguier P, Gourbeyre E, Chandler M. 2014. Bacterial insertion sequences: their genomic impact and diversity. FEMS Microbiol Rev 38:865–891. doi:10.1111/1574-6976.12067. 24499397 PMC7190074

[17] Vandecraen J, Chandler M, Aertsen A, Van Houdt R. 2017. The impact of insertion sequences on bacterial genome plasticity and adaptability. Crit Rev Microbiol 43:709–730. doi:10.1080/1040841X.2017.1303661. 28407717

[18] Yoon E-J, Goussard S, Touchon M, Krizova L, Cerqueira G, Murphy C, Lambert T, Grillot-Courvalin C, Nemec A, Courvalin P. 2014. Origin in Acinetobacter guillouiae and dissemination of the aminoglycoside-modifying enzyme Aph (3′)-VI. mBio 5:e01972-14. doi:10.1128/mBio.01972-14. 25336457 PMC4212838

[19] Ballouz S, Francis AR, Lan R, Tanaka MM. 2010. Conditions for the evolution of gene clusters in bacterial genomes. PLoS Comput Biol 6:e1000672. doi:10.1371/journal.pcbi.1000672. 20168992 PMC2820515

[20] von Wintersdorff CJ, Penders J, van Niekerk JM, Mills ND, Majumder S, van Alphen LB, Savelkoul PH, Wolffs PF. 2016. Dissemination of antimicrobial resistance in microbial ecosystems through horizontal gene transfer. Front Microbiol 7:173. doi:10.3389/fmicb.2016.00173. 26925045 PMC4759269

[21] Tanabe Y, Hodoki Y, Sano T, Tada K, Watanabe MM. 2018. Adaptation of the freshwater bloom-forming cyanobacterium Microcystis aeruginosa to brackish water is driven by recent horizontal transfer of sucrose genes. Front Microbiol 9:1150. doi:10.3389/fmicb.2018.01150. 29922255 PMC5996124

[22] Cohen KA, Manson AL, Desjardins CA, Abeel T, Earl AM. 2019. Deciphering drug resistance in Mycobacterium tuberculosis using whole-genome sequencing: progress, promise, and challenges. Genome Med 11:45. doi:10.1186/s13073-019-0660-8. 31345251 PMC6657377

[23] Malik T, Naim A, Saeed A. 2018. Molecular detection of TEM, SHV and CTX-M genes among Gram-negative Klebsiella isolates. Curr Drug Deliv 15:417–423. doi:10.2174/1567201815666180101160108. 29295691

[24] Conlon KM, Humphreys H, O'Gara JP. 2004. Inactivations of rsbU and sarA by IS256 represent novel mechanisms of biofilm phenotypic variation in Staphylococcus epidermidis. J Bacteriol 186:6208–6219. doi:10.1128/JB.186.18.6208-6219.2004. 15342591 PMC515138

[25] McEvoy CRE, Falmer AA, Gey van Pittius NC, Victor TC, van Helden PD, Warren RM. 2007. The role of IS6110 in the evolution of Mycobacterium tuberculosis. Tuberculosis (Edinb) 87:393–404. doi:10.1016/j.tube.2007.05.010. 17627889

[26] Tanaka MM, Rosenberg NA, Small PM. 2004. The control of copy number of IS 6110 in Mycobacterium tuberculosis. Mol Biol Evol 21:2195–2201. doi:10.1093/molbev/msh234. 15317877

[27] Pal C, Bengtsson-Palme J, Kristiansson E, Larsson DGJ. 2016. The structure and diversity of human, animal and environmental resistomes. Microbiome 4:54. doi:10.1186/s40168-016-0199-5. 27717408 PMC5055678

[28] Larsson DGJ, de Pedro C, Paxeus N. 2007. Effluent from drug manufactures contains extremely high levels of pharmaceuticals. J Hazard Mater 148:751–755. doi:10.1016/j.jhazmat.2007.07.008. 17706342

[29] Darby T, Jones R. 2017. Beneficial influences of Lactobacillus plantarum on human health and disease, p 109–117. *In* The microbiota in gastrointestinal pathophysiology. Elsevier, Oxford, United Kingdom.

[30] Zankari E, Hasman H, Cosentino S, Vestergaard M, Rasmussen S, Lund O, Aarestrup FM, Larsen MV. 2012. Identification of acquired antimicrobial resistance genes. J Antimicrob Chemother 67:2640–2644. doi:10.1093/jac/dks261. 22782487 PMC3468078

[31] Hyatt D, Chen G-L, LoCascio PF, Land ML, Larimer FW, Hauser LJ. 2010. Prodigal: prokaryotic gene recognition and translation initiation site identification. BMC Bioinformatics 11:119. doi:10.1186/1471-2105-11-119. 20211023 PMC2848648

[32] Buchfink B, Xie C, Huson DH. 2015. Fast and sensitive protein alignment using DIAMOND. Nat Methods 12:59–60. doi:10.1038/nmeth.3176. 25402007

[33] Bengtsson-Palme J, Larsson DGJ, Kristiansson E. 2017. Using metagenomics to investigate human and environmental resistomes. J Antimicrob Chemother 72:2690–2703. doi:10.1093/jac/dkx199. 28673041

[34] Pal C, Bengtsson-Palme J, Rensing C, Kristiansson E, Larsson DJ. 2014. BacMet: antibacterial biocide and metal resistance genes database. Nucleic Acids Res 42:D737–D743. doi:10.1093/nar/gkt1252. 24304895 PMC3965030

[35] Kurtz S, Phillippy A, Delcher AL, Smoot M, Shumway M, Antonescu C, Salzberg SL. 2004. Versatile and open software for comparing large genomes. Genome Biol 5:R12. doi:10.1186/gb-2004-5-2-r12. 14759262 PMC395750

[36] Liu M, Li X, Xie Y, Bi D, Sun J, Li J, Tai C, Deng Z, Ou H-Y. 2019. ICEberg 2.0: an updated database of bacterial integrative and conjugative elements. Nucleic Acids Res 47:D660–D665. doi:10.1093/nar/gky1123. 30407568 PMC6323972

[37] El-Gebali S, Mistry J, Bateman A, Eddy SR, Luciani A, Potter SC, Qureshi M, Richardson LJ, Salazar GA, Smart A, Sonnhammer ELL, Hirsh L, Paladin L, Piovesan D, Tosatto SCE, Finn RD. 2019. The Pfam protein families database in 2019. Nucleic Acids Res 47:D427–D432. doi:10.1093/nar/gky995. 30357350 PMC6324024

[38] Mistry J, Finn RD, Eddy SR, Bateman A, Punta M. 2013. Challenges in homology search: HMMER3 and convergent evolution of coiled-coil regions. Nucleic Acids Res 41:e121. doi:10.1093/nar/gkt263. 23598997 PMC3695513

[39] Li W, Godzik A. 2006. Cd-hit: a fast program for clustering and comparing large sets of protein or nucleotide sequences. Bioinformatics 22:1658–1659. doi:10.1093/bioinformatics/btl158. 16731699

[40] Siguier P, Pérochon J, Lestrade L, Mahillon J, Chandler M. 2006. ISfinder: the reference centre for bacterial insertion sequences. Nucleic Acids Res 34:D32–D36. doi:10.1093/nar/gkj014. 16381877 PMC1347377

[41] Oliphant TE. 2007. Python for scientific computing. Comput Sci Eng 9:10–20. doi:10.1109/MCSE.2007.58.

[42] Mitchell AL, Scheremetjew M, Denise H, Potter S, Tarkowska A, Qureshi M, Salazar GA, Pesseat S, Boland MA, Hunter FMI, Ten Hoopen P, Alako B, Amid C, Wilkinson DJ, Curtis TP, Cochrane G, Finn RD. 2018. EBI Metagenomics in 2017: enriching the analysis of microbial communities, from sequence reads to assemblies. Nucleic Acids Res 46:D726–D735. doi:10.1093/nar/gkx967. 29069476 PMC5753268

[43] Meyer F, Paarmann D, D'Souza M, Olson R, Glass EM, Kubal M, Paczian T, Rodriguez A, Stevens R, Wilke A, Wilkening J, Edwards RA. 2008. The metagenomics RAST server–a public resource for the automatic phylogenetic and functional analysis of metagenomes. BMC Bioinformatics 9:386. doi:10.1186/1471-2105-9-386. 18803844 PMC2563014

[44] Kristiansson E, Fick J, Janzon A, Grabic R, Rutgersson C, Weijdegård B, Söderström H, Larsson DGJ. 2011. Pyrosequencing of antibiotic-contaminated river sediments reveals high levels of resistance and gene transfer elements. PLoS One 6:e17038. doi:10.1371/journal.pone.0017038. 21359229 PMC3040208

[45] Pedregosa F, Varoquaux G, Gramfort A, Michel V, Thirion B, Grisel O, Blondel M, Prettenhofer P, Weiss R, Dubourg V. 2011. Scikit-learn: machine learning in Python. J Machine Learning Res 12:2825–2830.

