# Supplementary figures and images for: The Association between Insertion Sequences and Antibiotic Resistance Genes
Source: mSphere. 2020 Sep 2;5(5):e00418-20. doi: 10.1128/mSphere.00418-20 (PMC7471000; doi:10.1128/mSphere.00418-20)

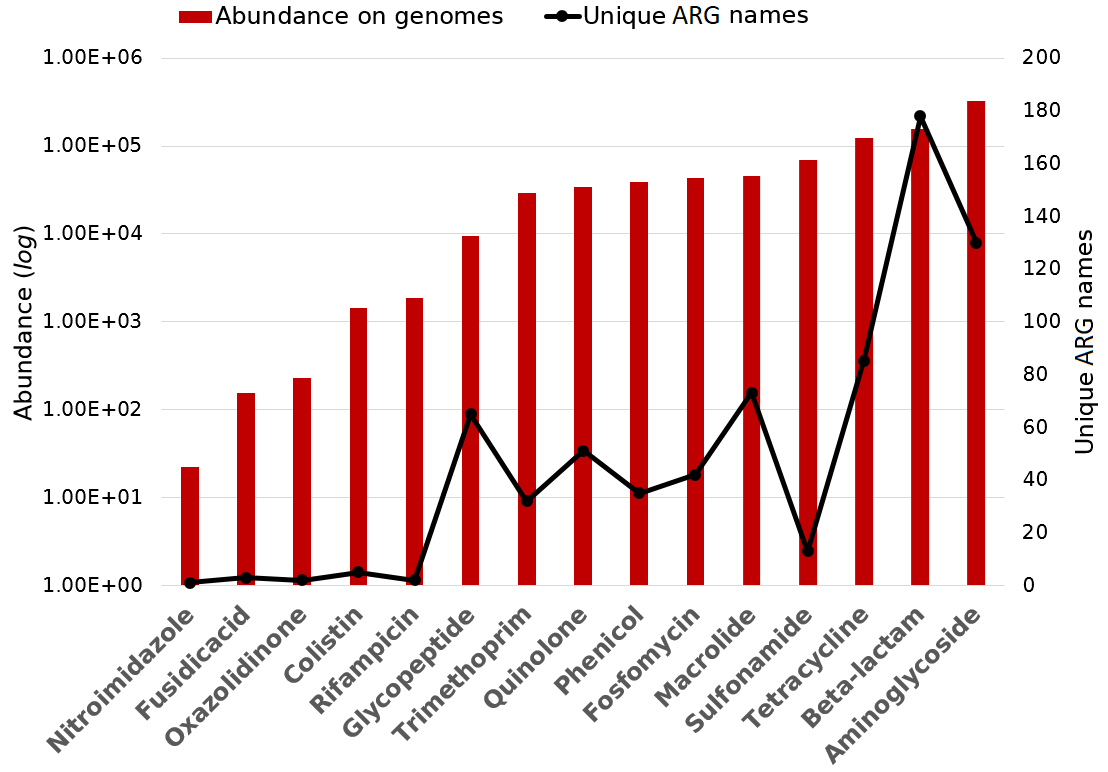

Supplement: FIG S1 [file mSphere.00418-20-sf001.tif]

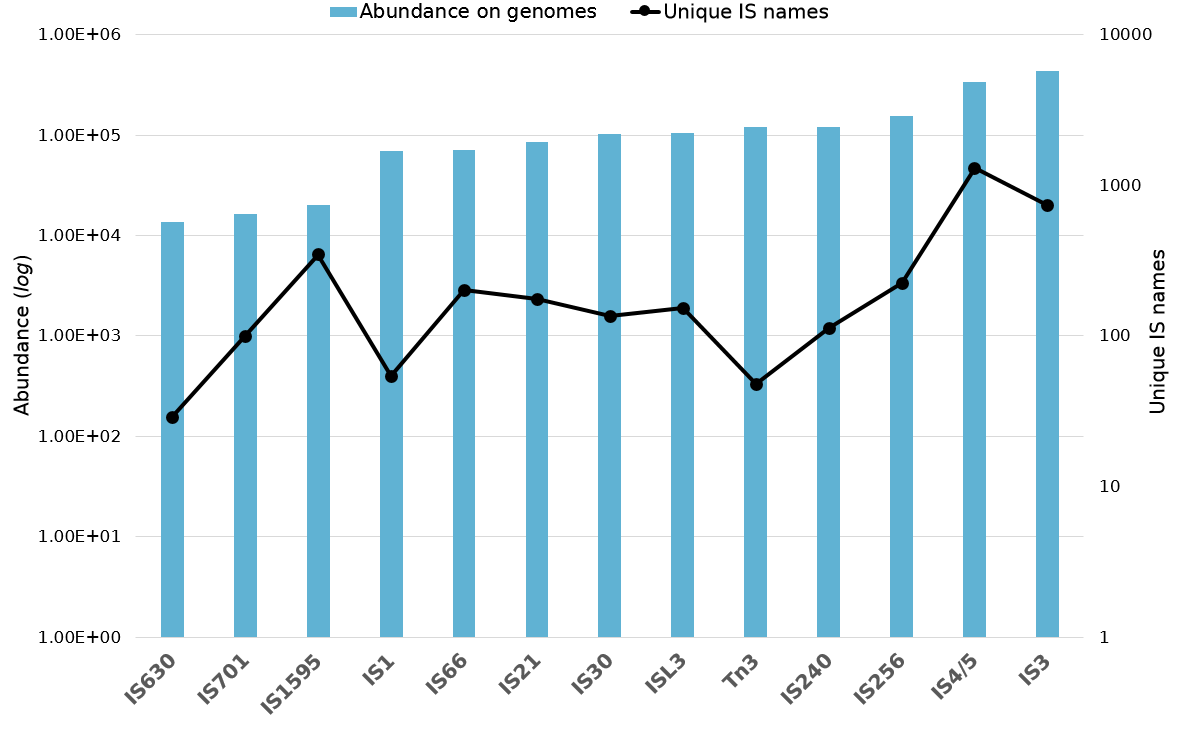

Supplement: FIG S2 [file mSphere.00418-20-sf002.tif]

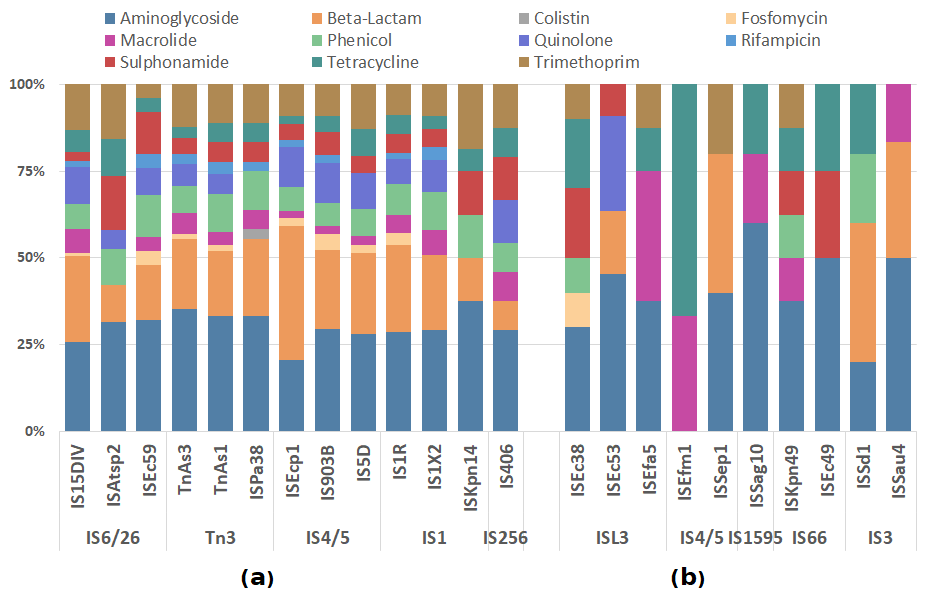

Supplement: FIG S3 [file mSphere.00418-20-sf003.tif]

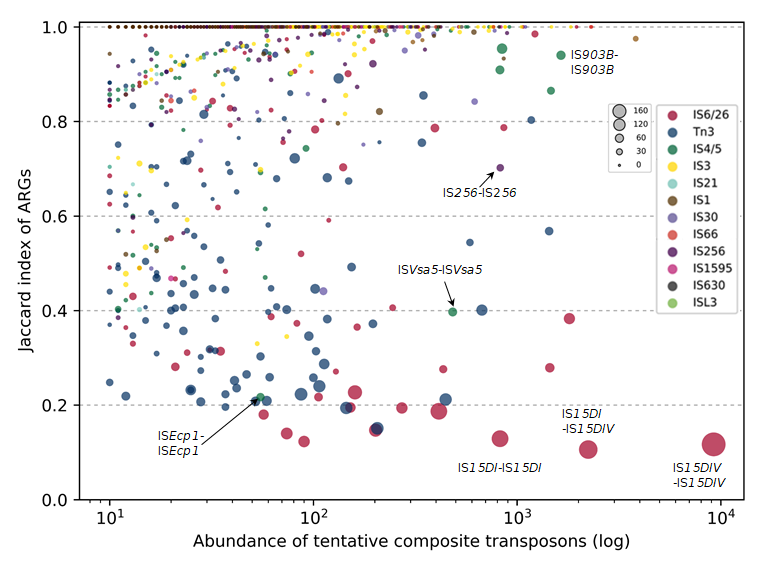

Supplement: FIG S4 [file mSphere.00418-20-sf004.tif]

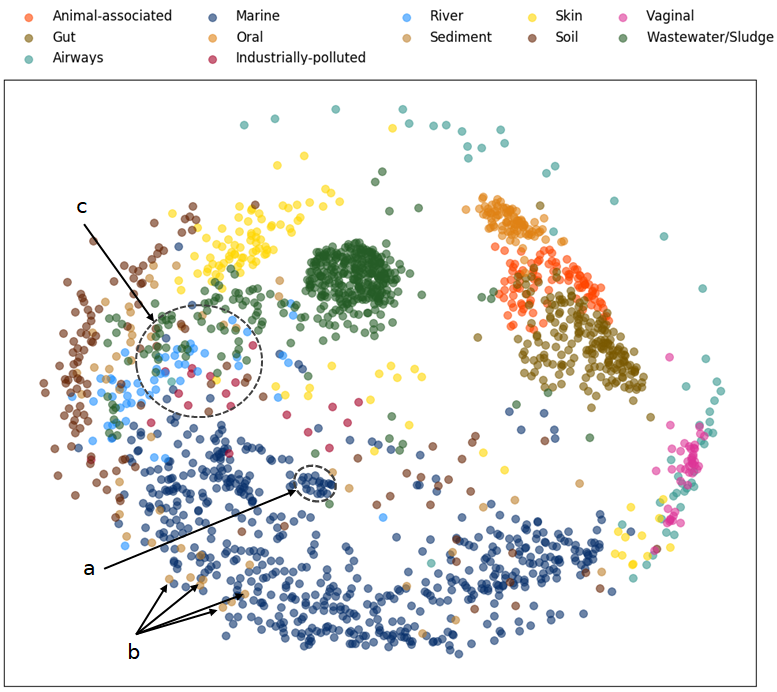

Supplement: FIG S5 [file mSphere.00418-20-sf005.tif]
