# Supplementary material for: The Association between Insertion Sequences and Antibiotic Resistance Genes
Source: mSphere. 2020 Sep 2;5(5):e00418-20. doi: 10.1128/mSphere.00418-20 (PMC7471000; doi:10.1128/mSphere.00418-20)
Supplement: TABLE S1 [file mSphere.00418-20-st001.docx]

Table S1. Proteins domains used to collect different IS elements

| **Alias** | **Domains ID** | **Domains Name** | **IS family** |
| --- | --- | --- | --- |
| IS*66* | PF03050 | DDE_Tnp_IS66 | IS*66* |
| IS*6/26* | PF13610 | DDE_Tnp_IS240 | IS*240*, IS*6*, IS*26* |
| IS*701* | PF13546 | DDE_5 | IS*701,ISH6* |
| IS*1* | PF03400 | DDE_Tnp_IS1 | IS*1* |
| IS*1595* | PF12762 | DDE_Tnp_IS1595 | IS*1595* |
| IS*4/5* | PF01609 | DDE_Tnp_1 | IS*4*, IS*421*, IS*5377*, IS*427*, IS*402*, IS*1355*, IS*5*, IS*1182*, IS*982*, IS*1634*, IS*1380* |
| IS*256* | PF00872 | Transposase_mut | IS*285*,IS*256* |
| IS*3* | PHA02517 | OrfB | IS*3* |
| ISL*3* | PF01610 | DDE_Tnp_ISL3 | ISL3, IS204, IS1001, IS1096 and IS1165 |
| Tn*3* | PF01526 | DDE_Tnp_Tn3 | Tn*3* |
| IS*630* | PF01710 | HTH_Tnp_IS630 | IS*630* |
| IS*30* | COG2826 | Tra8 | IS*30* |
| IS*21* | COG4584 | Transposase | IS*21* |
