# Supplementary material for: The Association between Insertion Sequences and Antibiotic Resistance Genes
Source: mSphere. 2020 Sep 2;5(5):e00418-20. doi: 10.1128/mSphere.00418-20 (PMC7471000; doi:10.1128/mSphere.00418-20)
Supplement: TABLE S2 [file mSphere.00418-20-st002.docx]

Table S2. Collection of metagenomic datasets that are used in this study

| **Environments** | **No. Metagenomes** | **No. Reads (Million)** |
| --- | --- | --- |
| **Marine** | 604 | 18634 |
| **Wastewater/Sludge** | 445 | 8648 |
| **Gut** | 200 | 4706 |
| **Oral** | 164 | 4083 |
| **Skin** | 147 | 2023 |
| **Soil** | 134 | 2244 |
| **Airways** | 110 | 4789 |
| **River** | 101 | 973 |
| **Animal-Associated** | 99 | 4294 |
| **Sediment** | 53 | 3078 |
| **Vaginal** | 50 | 2938 |
| **Industrially-polluted** | 21 | 472 |
